# GaAs Coupled Micro Resonators with Enhanced Sensitive Mass Detection

**DOI:** 10.3390/s141222785

**Published:** 2014-12-02

**Authors:** Tony Chopard, Vivien Lacour, Therese Leblois

**Affiliations:** 1 FEMTO-ST Institute, Université de Franche-Comté, 15B avenue des Montboucons, 25030 Besançon Cedex, France; E-Mails: tony.chopard@gmail.com (T.C.); vivien.lacour@usherbrooke.ca (V.L.); 2 Institute for Interdisciplinary Innovations in Technology (3IT), Faculty of Engineering, Université de Sherbrooke, 3000 Boulevard de l'Université, Sherbrooke, QC J1K OA5, Canada

**Keywords:** GaAs piezoelectric transducer, microcantilever, acoustic coupling, ultra-sensitivity, finite element method, biological sensing

## Abstract

This work demonstrates the improvement of mass detection sensitivity and time response using a simple sensor structure. Indeed, complicated technological processes leading to very brittle sensing structures are often required to reach high sensitivity when we want to detect specific molecules in biological fields. These developments constitute an obstacle to the early diagnosis of diseases. An alternative is the design of coupled structures. In this study, the device is based on the piezoelectric excitation and detection of two GaAs microstructures vibrating in antisymmetric modes. GaAs is a crystal which has the advantage to be micromachined easily using typical clean room processes. Moreover, we showed its high potential in direct biofunctionalisation for use in the biological field. A specific design of the device was performed to improve the detection at low mass and an original detection method has been developed. The principle is to exploit the variation in amplitude at the initial resonance frequency which has in the vicinity of weak added mass the greatest slope. Therefore, we get a very good resolution for an infinitely weak mass: relative voltage variation of 8%/1 fg. The analysis is based on results obtained by finite element simulation.

## 1. Introduction

Over recent decades there has been a growing interest in multiplexed, miniaturized, automated and cost-effective analytical techniques for environmental control, medical research and pharmacological screening. Low mass detectors are becoming highly desirable to detect small objects, like molecules or atoms [1–3]. For example, the field of biology requires mass detectors with a high sensitivity, especially in the range [1 zg, 10 fg], to detect the presence of particles (grafting onto a surface, deposition, adsorption). Some chemical and biochemical sensors have already demonstrated their suitability in these areas [4,5]. Recent advances in microfabrication technologies have triggered new applications for micro/nano tools. In particular, the ability to tailor the size and structure and hence the properties of micro/nano materials offers excellent prospects for designing novel sensing systems and enhancing the performance of the biochemical analytical assay. However, measurement mass techniques by conventional sensors require an important miniaturization to achieve sensitivity in the zeptogram range [1–8]. These manufacturing techniques remain complicated to develop and control. The main limitation for nanometer size devices is the fragility of these structures, especially in liquid environments. Coupled sensors networks do not require a high miniaturization, thus solving previous critical limitations, which explains that this concept aroused, since recently, notable enthusiasm [9–19]. Coupled structures also present other advantages such as the opportunity to perform on the same substrate differential measurements or to obtain a multiplexed analysis of a biological or chemical solution. Gallium arsenide material provides attractive properties for micro-biosensing due to the good current knowledge about its microfabrication technologies and opportunities for biofunctionalisation. In parallel, we proved the biocompatibility of GaAs surface for *in vitro* analysis [20–22]. The work presented here constitutes one of the preliminary steps necessary before the integration of electronics and transducer arrays on the same substrate for biological field measurements. The paper is divided into three sections. First, we present the principle of the coupled resonators and the usual method of mass measurement. Next we compare different methods of analysis to improve the sensitivity and time measurement. The results are obtained using a finite element model and the COMSOL Multiphysics^®^ software. Finally, we show the design of our device, describe the microfabrication process of the structure and present some preliminary results.

## 2. Measurement Principles

### 2.1. Theoretical Background

**Figure 1.**
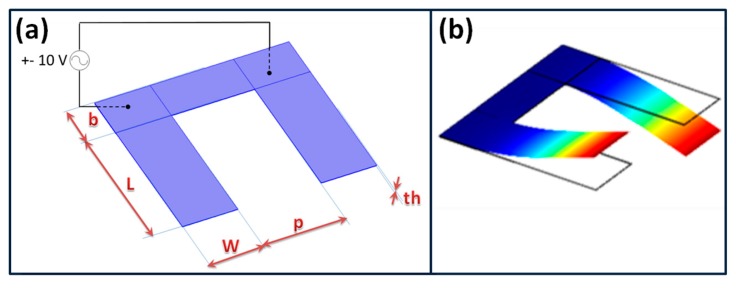
(**a**) Design of the coupled microcantilevers; (**b**) View of the first antisymmetric bending mode of vibration (color gives a qualitative indication of the displacement field).

**Figure 2.**
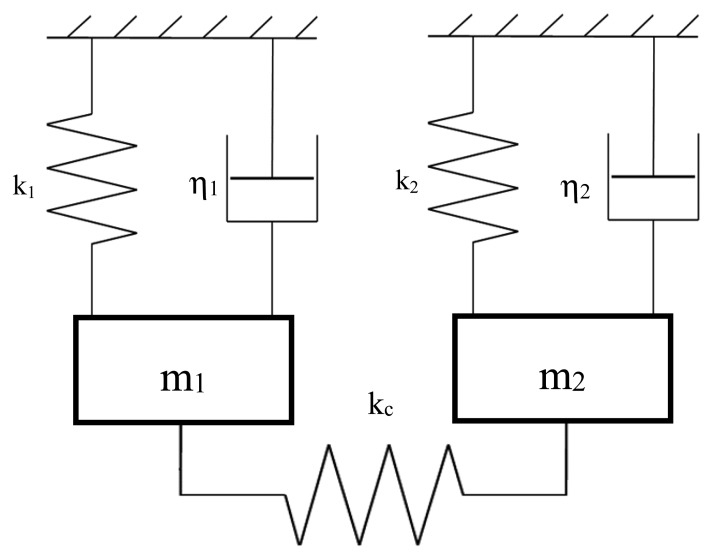
Schematic of the model of the coupled microcantilevers.

The current study is based on the microcantilever structures which have been previously demonstrated as suitable and inexpensive compared with other structures for biological field applications. Silicon, quartz or polymers are the most commonly used materials used in making microcantilevers, with typical dimensions ranging from tens to hundreds of micrometers long, widths of tens of micrometers, and hundreds of nanometers thick. In this study, cantilevers were made of GaAs which allows direct biofunctionalisation. A schematic of two identical cantilevers denoted as 1 and 2 and coupled by means of an overhang is shown in Figure 1a. The underlying physics of this system can be represented by the discretized model given in Figure 2. Each cantilever is modeled as a damped oscillator while the effect of the overhang coupling is modeled as a spring connecting the two oscillators. k_1_ and k_2_ are the bending stiffness of the oscillators and m_1_ and m_2_ are the suspended masses. Δ*m* is the added mass due to binding molecules. k_c_ is the stiffness of the overhang coupling of the two cantilevers. If we consider two identical cantilevers, the eigenvalues problem governing the undamped free oscillations of the device can be written as follows [19]:

(1) $$\left[\begin{array}{cc} 1-\varsigma & -\varsigma \\ -\varsigma & \left(1+\varsigma )/(1+\delta \right) \end{array}\right]\;u=\lambda u$$

where δ is the ratio of the effective mass added to the cantilever mass δ = Δ*m*/*m* and ζ is the ratio of the coupling stiffness to the cantilever stiffness k*_c_*/k*_i_*_ (_*_i_*_=1,2)_. λ is the eigenvalue and *u* the associated normalized eigenvector.

If *ζ* = 0, the eigenvalues and eigenvectors of the coupled cantilevers are:

(2) $$\lambda_{1}=1,\;{u_{1}}^{0}=\frac{1}{\sqrt{2}}\left[\begin{array}{c} 1\\ 1 \end{array}\right]\;\text{and}\;\lambda_{2}=1+2\zeta ,\;{u_{2}}^{0}=\frac{1}{\sqrt{2}}\left[\begin{array}{r} 1\\ -1 \end{array}\right]$$

The lower eigenvalue corresponds to the symmetric mode of vibration while the higher value corresponds to the antisymmetric mode. If ζ ≠ 0, eigenvalues and eigenvectors can be expressed as:

(3) $$\begin{array}{cc} \lambda_{i}=\lambda_{i}+\delta {\lambda_{i}}^{1}+\varepsilon (\delta^{2})\;\text{and}\;u_{i}=u_{i}+\delta {u_{i}}^{1}+\varepsilon (\delta^{2}) & i=1,2 \end{array}$$

Taking into account the Equation (3) it can be seen that the relative change in the eigenvectors is given below:

(4) $$\begin{array}{cc} \frac{\Delta u_{i}}{{u_{i}}^{0}}=\frac{\delta }{4\varsigma } & i=1,2 \end{array}$$

and the relative change in resonant frequency of the cantilever is:

(5) $$\frac{\Delta f_{R}}{{f_{R}}^{0}}=\frac{-\delta }{2}$$

In this study we consider the coupling cantilevers vibrating in an antisymmetric bending mode, whose amplitude variation is higher than the symmetric mode. The resonance frequency depends on the geometry of the two cantilevers and Young's modulus E. The geometry of the device is given in Figure 1a. With the following dimensions L = 100 μm, w = 10 μm, th = 2 μm, p = 20 μm and b = 20 μm, the first antisymmetric mode (Figure 1b) resonance frequency *f_R_* is obtained at *f_R_* = 109.468 kHz.

### 2.2. Model Procedures

The sensor structures were created and simulated using a finite element modeling (FEM) tool (MEMS module) of COMSOL Multiphysics^®^ 3.5a (COMSOL Inc., Stockholm, Sweden) to study the resonant characteristics and the sensitivity of the device for femtogram mass detection. Three analyses were used: static, eigenfrequency and transient/time-dependent. The static analysis was used to find the magnitudes and location of maximum stresses/strain and electrical potential at several points of the cantilever when a static load was applied to the beam's free end. The eigenfrequency analysis was performed to determine the first modes of vibration and the associated mode shapes (flexion, torsion, elongation). Finally, time-dependent analysis was carried out to solve the transient solution when the applied load was time-dependent. The components of stiffness and piezoelectric GaAs tensors were introduced in the library. The damping coefficient of GaAs material which was estimated from experimental Q factors was introduced in the library. It was estimated at η =10^−5^. 3D modeling was used in this problem. The structures were meshed using quadratic Lagrange elements. The Sparse Object-Oriented Linear Equations Solver (library SPOOLES [23]) was used to obtain the results of simulation. Several scripts were written with Matlab^®^ (MathWorks, Inc., Natick, MA, US) to optimize and facilitate the analysis of results.

### 2.3. Conventional Measurements Methods

The frequency response is measured to identify the maximum amplitude of the resonance peak, before and after the addition of mass. The usual method consists of the experimental determination of the frequency shift Δ*f_R_* of the resonance frequency *f_R_* with the added mass Δ*m*. The expression for Δ*f_R_* is given below in Equation (5). It depends on the resonator mass *m* and an increased mass sensitivity can be achieved by decreasing mass, so the variation of amplitude Δ*u_i_* is a quasi-linear function of the added mass Δ*m* given by Equation (4). This last method is preferred to the frequency shift determination when ζ < 1/2. We performed calculation on an antisymmetric bending mode. Two methods can be used to determine the added mass:

(a) Method A: determination of the frequency shift Δ*f_R_* with the added mass.
(b) Method B: determination of the amplitude change Δ*u_2_* at the resonance peak by frequency sweeping with the added mass.

**Figure 3.**
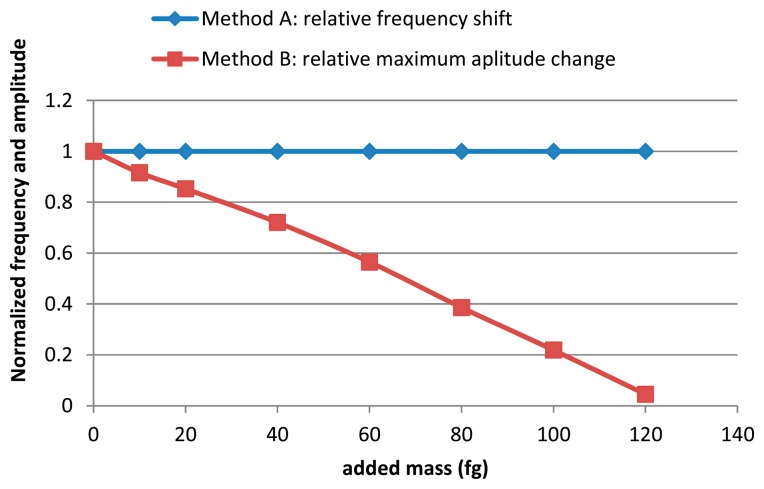
Comparison between the relative change in resonance amplitude and the relative change in resonance frequency *versus* added mass.

Like in the publication of Gil-Santos *et al.* [12], we observed a quasi-linear decrease of the amplitude as a function of the added mass. We obtained in the range [0, 150 fg], a linear relative variation in resonance peak amplitude of 8000 ppm/fg against 2 ppm/fg for the relative variation in resonance frequency. These results are shown in Figure 3. These values confirm the advantages of the amplitude method we have called method B on coupled microcantilevers.

Nevertheless the sensitivity is insufficient to detect biological or chemical molecules. We propose to improve the performance by using different method to exploit the frequency *versus* mass spectrum.

### 2.4. Enhanced Measurement Methods

**Figure 4.**
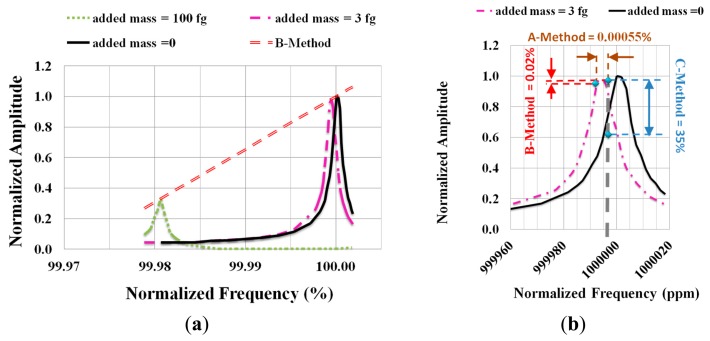
(**a**) Amplitude with and without added mass vs resonance frequency; (**b**) Zoom on the overlap shown in (a).

As seen on Figure 4a we have a resonance peak overlap for a very weak added mass and so it remains difficult to reach the required sensitivity. To improve performances using the same measurements principle as previously, we propose, for very weak mass, to determine the variation of the amplitude at the fixed frequency *f_R_*. *f_R_* is the resonant frequency at the initial weight *m*. This method is called method C. Compared to methods A and B, a significant increase of the relative variation in the amplitude is obtained (see Figure 4b).

Moreover, as the frequency sweep can be omitted, the measurement process is easier and the acquisition time is significantly reduced. This latter criterion may be a key point for the determination of parameters in a biological analysis.

It is clear that the higher the quality factor, the higher the sensitivity of the transducer. The operating range which is one of the criteria for transducer characterization is given by Equation (6). It can be deduced from the initial microcantilever mass *m*, the resonance frequency of the selected mode *f_R_* and the half-width of the resonance peak at 10% of the relative variation in amplitude:

(6) $$\text{Range}\approx \frac{m\times \Delta f_{R(\Delta \text{relative amplitude change}=10\%)}}{f_{R}}$$

**Figure 5.**
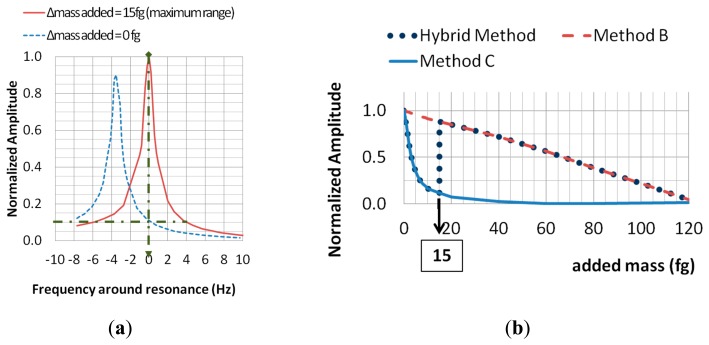
(**a**) Amplitude *versus* frequency: determination of the operating range; (**b**) Definition of hybrid method.

Method C is valid as long as there is a monotonic overlap of the resonance peak with added mass and the resonance characteristic at initial weight. In the case of Figure 5a, we have Δ*f_R_*_(_*_Δrelative amplitude change_*_ = 10%)_ = 4 Hz and the operating range is equal to 15 fg. It may be noticed that if the resonance curve has a better quality factor, then the resolution increases but the operating range decreases.

As the operating range is small, we propose to use a hybrid method to exploit the good performances of C-method when we are concerned with very weak added mass (see Figure 5b). Over the operating range without modifying the device we propose to use method B. With our device, the threshold for switching between the two methods is for Δ*m* = 15 fg, which corresponds to a relative variation in the maximum deflection of 11.5%.

### 2.5. Device Design

**Figure 6.**
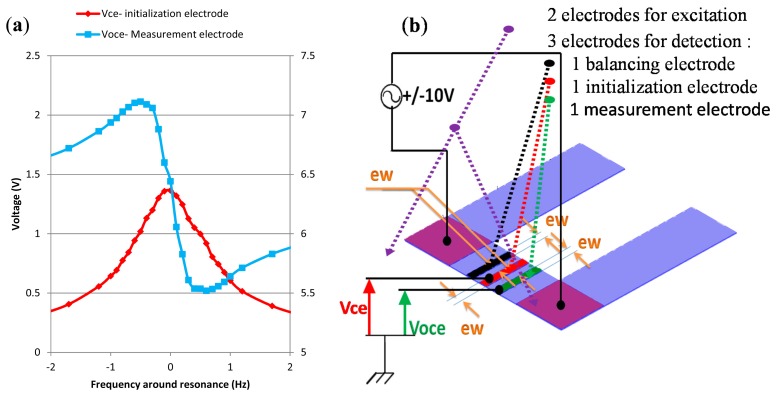
(**a**) Frequency analysis of voltage signals Vce and Voce; (**b**) Piezoelectric excitation and detection with three electrodes placed at regular intervals and centered on the coupling element.

To adapt the previous methods we used a device with a piezoelectric excitation and detection. Unlike optical methods piezoelectric ones allow measurement of resonance peaks with the appearance of an even or odd differentiable function. The appearance depends on the electrode position on the overhang. According to a study parameterized in frequency on the voltage change within the coupling element, we determined that we need to deposit an electrode at the coupling element center of the microcantilevers. The electrode is a gold layer which thickness is 50 nm and area (b × ew = 8 μm × 1.5 μm). The signal delivered by this electrode is an even function. Nevertheless, an off center electrode provides an odd function. As shown in Figure 6b, the device is composed of three electrodes. The balancing electrode helps keeping the symmetry of the device.

With this device, we obtained in the range from 1 zg to 0.1 fg a resolution 36 times higher than with method B. This corresponds to a relative variation in voltage of 8%/fg which is a very good sensitivity for the infinitely weak mass according to the size of the device. To expand the operating range we used as already seen the hybrid method of analysis.

## 3. Microfabrication Processes of the Device and Preliminary Results

### 3.1. Device Microfabrication Process

The microfabrication process was designed to manufacture a reproducible device at low cost. The process used photolithography and wet anisotropic chemical etching processes. Table 1 gives some advantages and disadvantages of chemical etching technique and GaAs substrates for this application. The use of cantilever as sensing structure is also discussed. The microfabrication and characterization were performed at the clean room (MIMENTO).

**Figure 7.**
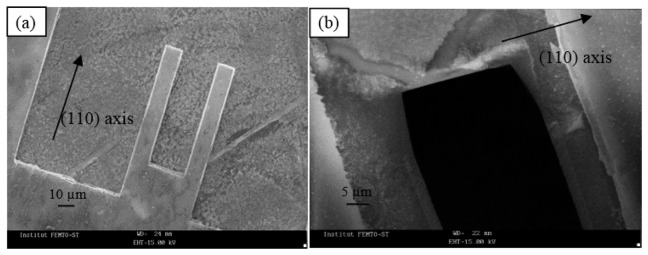
SEM images of chemically etched microcantilevers. The cantilever is covered by a chemically inert mask: (**a**) coupled cantilevers; (**b**) magnification of the structure: underetching under the mask.

Small samples were cut into 500 μm squares by sawing from a 600 μm thick wafer of GaAs (wafer diameter of 3 inches). Prior to the deposition of gold/chromium electrodes, the GaAs surface was deoxidized with an HCl based solution. Gold/chromium layers were deposited on the upper face of the wafer by sputtering and lift-off technique was used for electrodes patterning. A chemical thinning process is applied to the sample using 7 H_3_PO_4_:5 H_2_O_2_:8 H_2_O solution [26] and the temperature of the etchant was maintained at 0 °C ± 0.2 °C for an efficient control of the etching depth. After this step the sample thickness was 20 μm. A photolithographic process followed by etching was performed. It was carried out using two successive wet etchings with an orthophosphoric acid-based solution 1 H_3_PO_4_:9 H_2_O_2_:1 H_2_O maintained at 0 °C ± 0.2 °C with ultrasonic stirring. The first one allowed thickness control and the second one was used to make the cantilevers. The choice of wet etching baths and the etching conditions are detailed in [26]. Figure 7 gives SEM views of the cantilevers that were obtained after chemical etching. In the SEM image, the inert mask covered the surface of the device. Figure 7a shows the device whereas Figure 7b gives information on the shape under the mask near the clamping positions of the beams. The analysis of etching shapes revealed a large underetching under the mask and convex undercutting at the free end of the cantilever. The lateral sides of the beams are not constituted by vertical walls but by inclined blocking facets and the cross sections of the beam are then not squares.

### 3.2. Preliminary Results

The structures of the coupled microcantilevers were characterized using optical microscope and SEM just after chemical etching. The mask was then removed and the coupled microcantilevers were electrically tested using an impedance network analyzer. To characterize the device with an added mass, we deposited successive Si_3_N_4_ layers on GaAs by low temperature ICPECVD (Plasma deposition system SI 500 D from SENTECH). We added two layers of 10 nm ± 1 nm of Si_3_N_4_ and we made electrical measurements between each deposition. Each layer had an approximate mass of 0.2 μg (ρ = 1000 kg·m^−3^). Table 2 gives some preliminary results.

In the table, we observe a significant difference between experimental and theoretical results. These differences concern the dimensions of the structure and also the mass measurements. The shift in geometrical values L, b, p, w and th is due to undercutting. This unwanted effect occurs in particular in the fabrication of very thin devices. Undercutting can be reduced or even prevented by corner compensation structures [27] which are added to the corners in the mask layout. The change in size and shape of the structure induces a shift in frequency (Δf_R_/f_R_ = 21.8% as seen in Table 2) which can be easily explained. Nevertheless, the experimental resonant frequency in bending antisymmetric mode is in agreement with the theoretical value.

As concern mass measurements, we were not able to obtain results with method C because the added masses were out of the operating range. Complementary tests with lower added masses must be carried out. With regard to the relative variation of frequency (method A), experimental and theoretical results are close. The relative errors can be easily explained (i) by the change in size and shape between simulation and experimental results; (ii) by the error in Si_3_N_4_ thickness films (ii) by the uncertainty in the density of Si_3_N_4_ films which induces uncertainty in the added mass. Complementary measurements on films structures and properties have to be done to improve the quality of results. The order of magnitude of the sensitivity with regard to the added mass is the same but as already said the sensitivity is too low for biological sensing (required sensitivity < pg/mm^2^). For the relative variation of amplitude (method B) experimental and theoretical values are similar and these results confirm the enhancement of the sensitivity with this method.

## 4. Conclusions/Outlook

To conclude, we have shown that the sensitivity of mass sensors based on coupled cantilevers can be significantly enhanced by a thorough analysis of the measurement and an original design of structures and electrodes. We have proposed and demonstrated the benefit of an analysis method which significantly increases the resolution for the addition of very weak mass on coupled microcantilevers. Moreover, utilizing such a method for mass detection offers a real advantage over the more conventional resonant frequency shift approach and the maximum amplitude method. Indeed, thanks to this attractive analysis method, the fabrication of highly miniaturized devices which are brittle can be avoided. Optical and piezoelectric detections have been considered and compared in terms of sensitivity at very weak added mass. The proposed method is particularly efficient for piezoelectric detection using appropriate electrode designs. The theoretical results with this method are promising and the device remains easy to fabricate. The low cost microfabrication process in piezoelectric GaAs crystal is relevant. Preliminary tests were engaged to characterize the coupled device. The preliminary electrical results are promising. Additional measurements are needed to prove the relevance of the device and the relevance of the methods of detection designated as B and C. In the future, first we like to achieve coupled sensors whose structure is based on membranes instead of beams to change the mode of vibration, increase the resonant frequency and improve the quality factor. Second we want to increase the number of coupled resonators in order to obtain for biological applications in addition to the high sensitivity the opportunity of multiplexed measurements.

## Figures and Tables

**Table 1.** Advantages and disadvantages of GaAs wet chemical etching of cantilevers.

Wet chemical etching	Advantages: - Mass production - Low cost equipment as compared with RIE system - Process with a high level of repeatability - High etched rates - High anisotropy of etch rates

	Disadvantages: - Final shape governed by the crystallographic planes - Low etch factor as compared with RIE - Limitation in the fabrication of 3D structures at nanometer size: dry etching is preferred [24]

GaAs substrate	Advantages: - Efficiency of the thiolate functionalisation on GaAs surface - Integration of electronics - Piezoelectric material with a high coupling factor - High quality of material - Good mechanical properties - Clean room processes already developed

	Disadvantages: - Anisotropy in chemical etching less marked than for silicon - GaAs wafers more expensive than silicon wafers

	Advantages: - Easy to fabricate and to functionalize - Low resonant frequencies easy to analyze - sensitive for biological applications [24]

	Disadvantages: - High deflections of the beam - Decrease of Q factor higher for cantilever in flexural or torsion mode than for plate vibrating in thickness shear mode (QCM) in liquid [25]

**Table 2.** Preliminary results of coupled microcantilevers. Comparison with theoretical results.

**Electrical and Geometrical Results**	**f_R_ (kHz)**	**Q factor**	Method A (Δf_R_/f_R_) *	Method B (ΔA/A) *	**Length (μm)**	**Width (μm)**	**Thickness (μm)**	**Overhang b (μm)**	**Gap p (μm)**
Experimental results	133.421	18,460	4.10^−4^	1.6	95	11	2.2	21	22
			8.10^−4^	3.2					

Theoretical results	109.468	25,000	5.10^−4^	2.5	100	10	2	20	20
			11.10^−4^	2.7					

Relative error (%)	21.8	26.1	25	56	5	10	10	5	10
			37	15					

* The results are given for the two successive depositions of Si_3_N_4_ films.

## References

[1] Battiston F.M., Ramseyer J.P., Lang H.P., Baller M.K., Gerber Ch., Gimzewski J.K., Guntherodt H.J. (2001). A chemical sensor based on a microfabricated cantiler array with simultaneous resonance-frequency and bending readout. Sens. Actuators B Chem..

[2] Rogers B., Manning L., Jones M., Sulcheck T., Murray K., Beneschott B., Adams J.D., Hu Z., Thundat T., Cavazos H. (2003). Mercury vapor deposition with a self-sensing resonating piezoelectric cantilever. Rev. Sci. Instrum..

[3] Mertens J., Finot E., Nadal M.H., Eyraud V., Heintz O., Bourillot E. (2004). Detection of gas trace of hydrofluoric acid using microcantilever. Sens. Actuators B Chem..

[4] Hosaka H., Chiyoma T., Ikeuchi A., Okano H., Sone H., Izumi T. (2006). Possibility of a femtogram mass biosensor using a self-sensing cantilever. Curr. Appl. Phys..

[5] Wang C., Wang D., Mao T., Hu X. (2007). Ultrasensitive biochemical sensors based on microcantilevers of atomic force microscope. Anal. Biochem..

[6] Yang Y., Callegari C., Feng X., Ekinci K., Roukes M. (2006). Zeptogram-scale nanomechanical mass sensing. Nano Lett..

[7] Jensen K., Kim K., Zettl A. (2008). An atomic resolution nanomechnical mass sensor. Nat. Nanotechnol..

[8] Gil-Santos E., Ramos D., Martinez J., Fernandez-Regulez M., Garcia R., San Paulo A., Calleja M., Tamayo J. (2010). Nanomechnanical mass sensing and stiffness spectrometry based on two-dimensional vibrations of resonant nanowires. Nat. Nanotechnol..

[9] Hansen K.M., Thundat T. (2005). Microcantilever biosensors. Methods..

[10] Spletzer M., Raman A., Sumali H., Sullivan J. (2008). Highly sensitive mass detection and identification using vibration localization in coupled microcantilever arrays. Appl. Phys. Lett..

[11] De Martini B.E., Rhoads J.F., Shaw S.W., Turner K.L. (2007). A single inputs-single output mass sensor based on a coupled array of microresonators. Sens. Actuators A Phys..

[12] Thiruvenkatanathan P., Yan J., Woodhouse J., Seshia A. (2009). Enhancing parametric sensitivity in electrically coupled MEMS resonators. J. Microelectromech. Syst..

[13] Karabalin R., Cross M., Roukes M. (2009). Nonlinear dynamics and chaos in two coupled nanomechanical resonators. Phys. Rev. B.

[14] Okamoto H., Kitajima N., Onomitsu K., Kometani R., Warisawa S., Ishihara S., Yamaguchi H. (2011). High sensitivity charge detection using antisymmetric vibration in coupled micromechanical oscillators. Appl. Phys. Lett..

[15] Gil-Santos E., Ramos D., Pini V., Calleja M., Tamayo J. (2011). Exponential tuning of the coupling constant of coupled microcantilevers by modifying their separation. Appl. Phys. Lett..

[16] Huber T.M., Abell B.C., Mellema D.C., Spletzer M., Raman A. (2010). Mode-selective non-contact excitation of microcantilevers and microcantilever arrays in air using the ultrasound radiation force. Appl. Phys. Lett..

[17] Chopard T., Bienaime A., Elie-Caille C., Leblois T. (2012). High sensitive mass detection using piezoelectric coupled microcantilevers. Procedia Engineer..

[18] Thiruvenkatanathan P., Yan J., Woodhouse J., Aziz A., Seshia A. (2010). Ultrasensitive mode-localized mass sensor with electrically tunable parametric sensitivity. Appl. Phys. Lett..

[19] Spletzer M., Arvind R., Wu A.Q., Xu X. (2006). Ultrasensitive mass sensing using mode localization in coupled microcantilevers. Appl. Phys. Lett..

[20] Bienaime A. (2011). Microcapteur GaAs pour la Détection de Molécules Dans un Fluide Biologique. Ph.D. Thesis.

[21] Dubowski J.J., Voznyy O., Marshall G.M. (2010). Molecular Self-assembly and Passivation of GaAs(001) with Alkanethiol Monolayers: A View Towards Bio-functionalization. Appl. Surf. Sci..

[22] Bienaime A., Leblois T., Gremaud N., Chaudon M.J., el Osta M., Pecqueur D., Ducoroy P., Elie-Caille C. (2013). Influence of a thiolate chemical layer on GaAs(100) biofunctionalization: An original approach coupling AFM and mass spectrometry methods. Materials.

[23] Linalg/Spooles. www.netlib.org/linalg/spooles/.

[24] Morris D.R., Fatisson J., Olsson A.L.J., Tufenkji N., Ferro A.R. (2014). Real-time monitoring of airborne cat allergen using a QCM-based immunosensor. Sens. Actuators B Chem..

[25] Wasisto H.S., Merzsch S., Stranz A., Waag A., Uhde E., Salthammer T., Peiner E. (2013). Femtogram aerosol nanoparticle mass sensing utilizing vertical silicon nanowire resonators. Micro Nano Lett..

[26] Bienaimé A., Elie-Caille C., Leblois T. (2012). Microstructuration of GaAs surface by wet etching: Towards a specific surface behavior. J. Nanosci. Nanotechnol..

[27] Zhang Q., Liu L., Li Z. (1996). A new approach to convex corner compensation for anisotropic etching of (100) Si in KOH. Sens. Actuators A Phys..

